# “I Want to Spend My Time Living”—Experiences With a Digital Outpatient Service With a Mobile App for Tailored Care Among Adults With Long-Term Health Service Needs: Qualitative Study Using Thematic Analysis

**DOI:** 10.2196/79155

**Published:** 2026-01-15

**Authors:** Heidi Holmen, Erik Fosse

**Affiliations:** 1 The Intervention Centre Oslo University Hospital Oslo Norway; 2 Department of Nursing and Health Promotion Faculty of Health Sciences OsloMet—Oslo Metropolitan University Oslo Norway; 3 Institute of Clinical Medicine Faculty of Medicine University of Oslo Oslo Norway

**Keywords:** digital outpatient care, mHealth, patient-centered care, patient experiences, digital outpatient services

## Abstract

**Background:**

Digital health services are increasingly used in hospital-based outpatient care, offering remote monitoring, patient-reported outcomes, information sharing, and asynchronous communication. While expected to improve self-management, timeliness, and efficiency, the success of digital health interventions relies on patients’ health literacy and digital health literacy. While some research has addressed potential associations between digital health interventions and patients’ health outcomes, research on patients’ experiences remains limited.

**Objective:**

The aim of this study was to explore and gain in-depth knowledge about the experiences of patients with chronic or long-term conditions enrolled in a 6-month digital outpatient care intervention for tailored care and health literacy.

**Methods:**

We conducted an exploratory qualitative interview study with 17 strategically recruited adult patients with cancer, interstitial lung disease, epilepsy, or complicated pain who used a digital outpatient service for 6 months. Individual telephone interviews were conducted using a semistructured guide, transcribed verbatim, and analyzed with thematic analysis to generate codes and themes. Participants had a median age of 62 years (minimum-maximum 36-83 years), with 8 females and 9 males.

**Results:**

The thematic analysis led to 1 main theme “Digital outpatient care as a flexible service supporting patients’ self-management,” informed by 3 subthemes “The ongoing nature of managing a chronic condition and how the digital service meet the patients’ desire for autonomy in their care,” “Digital tools flexibly address the patients’ unique needs, but reliability depends on patient interaction,” and “Digital services enhance the patients’ sense of safety through easy access to a relation with competent healthcare workers.” The themes highlight patients’ appreciation for greater flexibility in their care and their desire to self-manage with the support of easily accessible health care workers, ultimately supporting their health literacy. Patients recognized the importance of actively engaging with the digital solution to fully benefit from its opportunities and emphasized the critical role of health care workers in fostering their sense of security.

**Conclusions:**

Digital outpatient care was experienced as flexible and supportive for patients with long-term conditions. The increased possibility of interacting with health care workers was welcomed by the patients, and the combination of flexibility, self-monitoring, and addressing concerns regarding their self-management may increase the patients experience of autonomy. As health literacy likely plays a role in patients’ ability to effectively engage with digital tools and self-manage their conditions, future research should explore how varying levels of health literacy influence these outcomes. In addition, research should address whether such digital outpatient clinics are positive for a wider range of patients, associated health outcomes, and any positive effects on a health system level.

**Trial Registration:**

ClinicalTrials.gov NCT05068869; https://clinicaltrials.gov/ct2/show/NCT05068869

## Introduction

The increasing demand for health care services is not matched by available resources, including workforce shortages [1,2]. To enhance the sustainability of health services, greater flexibility is needed to address the dynamic needs of patients with long-term conditions, aligning with patient-centered care and optimizing resource use. Digital tools have been proposed as a means to achieve this flexibility, offering functionalities such as patient monitoring, self-reporting of objective and subjective data, asynchronous communication, and video consultations [3]. These tools can help identify patients at risk for deterioration or in need of immediate care, while also detecting those stable enough to delay scheduled services. By supporting patient-centered care, digital health solutions may improve attendance at scheduled appointments, enhance symptom management, and empower patients in self-management.

Digital health care services typically enable the subjective reporting of health parameters through patient-reported outcome measures [4]. These measures can aid self-management and support health literacy [5] and facilitate communication between patients and health care workers [6]. Furthermore, digital engagement, self-monitoring, and data sharing offer several advantages for patients [3,7,8]. Recent studies suggest that digital tools in outpatient care for patients with long-term conditions, such as cancer, epilepsy, interstitial lung disease, and musculoskeletal pain, can prevent complications, encourage patient engagement, and increase confidence and autonomy [9-12]. However, research on multicomponent digital solutions—encompassing patient-reported outcome measures, asynchronous messaging, remote monitoring, patient notifications, and video consultations—is limited. In addition, one important prerequisite exists for patients to benefit from digital health services, namely, that the patients use the services as intended. Patients’ use of a digital health services might rely on their health literacy and ability to self-manage, and a certain level of digital skills and digital health literacy is also needed [5]. Health literacy provides a theoretical lens for this study, as it is a foundational factor influencing how patients access, understand, and use digital health services. Defined as “the cognitive and social skills that determine the motivation and ability of individuals to gain access to, understand, and use information in ways which promote and maintain good health” [13], health literacy not only affects patients’ capacity for self-management but also plays an integral role in their engagement with digital tools. Often, higher health literacy is associated with an increased benefit of digital health interventions [14], while lower health literacy is linked to poorer health outcomes and less digital solution usage [15,16]. Furthermore, adequate health literacy is key to optimizing the use of digital health solutions, as it is suggested to enhance self-management in chronic conditions [5,16-18]. Although several systematic reviews have explored how to define, measure, and understand health literacy and digital health literacy [17,19-24], linking this knowledge to the experience of patients participating in digital health interventions remains scarce. Digital health literacy has been suggested as a super determinant of health comprising more than simply digital literacy and health literacy [25]; thus, exploring the experiences of patients with a digital health intervention supporting flexibility and health literacy might contribute with knowledge on how to research and design more effective, inclusive, and patient-centered digital health solutions that address diverse needs and promote equitable health outcomes.

While research on digital health services expands, research on patients’ experiences of participating and making use of integrated digital solutions in outpatient care is lacking. The meaningfulness of a digital health solution depends on factors such as its usability, clinical relevance, convenience, and evidence-based design [6]. Studies on video consultations suggest how they are more convenient and time-saving for patients, but they are not perceived as superior to traditional in-person consultations [26,27]. Thus, despite growing interest in specific features of digital outpatient care and to some extent, the combination of features, previous research remains limited concerning the patients’ experiences on a comprehensive model for digital outpatient care. By adopting health literacy as a guiding framework, the aim of this study was to explore and gain in-depth knowledge about the experiences of patients with chronic or long-term conditions enrolled in a digital 6-month outpatient care intervention.

## Methods

### Study Design

We conducted an explorative qualitative interview study as part of a larger study evaluating the use of digital outpatient care for adults with cancer, interstitial lung disease, epilepsy, or a complex pain condition at 2 university hospitals in Norway [28,29]. The study is reported according to the COREQ (Consolidated criteria for Reporting Qualitative research) guidelines [30].

### Setting

The setting for this study was the 4 outpatient services participating in a multicenter controlled trial evaluating digital outpatient services, comprising the Department of Respiratory Diseases, the Department of Neurology, and the Department of Pain Management at Oslo University Hospital, as well as the Department of Cancer at the University Hospital of North Norway [28,29]. All patients included in this trial were living at home, receiving outpatient care, and otherwise managing their own lives.

Eligible participants had been included in the digital outpatient intervention, previously detailed [28,29]. The core focus of the intervention was to improve outpatient service accessibility, allowing the patients to engage with patient-reported outcome measures, self-monitor parameters, and communicate asynchronously with health care workers. Designated health care workers assessed the patients’ responses and parameters and had asynchronous digital contact, aligning with a conceptual model linking health literacy to service access and self-management [31]. Health care workers assigned tasks to patients and set individual thresholds using a traffic light model. Notifications alerted health care workers of deviations such as increased pain, side effects, or uncompleted tasks. Patients received reminders for unfinished tasks. Two-way messaging enabled asynchronous communication for questions, information exchange, and sharing of treatment plans. The digital outpatient intervention was enabled through the Dignio Connected Care platform, allowing personalized patient–health care worker contact [32] (Multimedia Appendices 1 and 2). The platform, including the health care worker’s Dignio Prevent system and the patient’s MyDignio app, is Conformité Européenne marked, privacy compliant, and globally applied [32].

### Recruitment and Study Participants

Patients in the intervention arm of the overall multicenter controlled trial were invited to be interviewed when they completed their 6-month follow-up questionnaire at the end of the digital intervention. One item of the questionnaire asked for their consent to be contacted by a researcher with information regarding the qualitative interviews. An a priori estimation suggested a sample of 12-15 patients from the intervention group [28]. A strategic recruitment among those accepting to be contacted was conducted to ensure a broad sample of interviewees, enabling our aim of collecting rich data on the experiences of using the digital outpatient care intervention. Oral information was given over the phone, prior to written information in a secure digital platform, also allowing for their digital consent. We made use of the secure service “Nettskjema” digital consent in a service for sensitive data (Tjeneste for Sensitive Data [TSD] in Norwegian: the English version is Service for Sensitive Data]) developed at the University of Oslo. TSD is designed for storing and processing sensitive data in compliance with the Norwegian “Personal Data Act” and “Health Research Act.” The consent invitations were sent to the participants through the Pretty Good Privacy encrypted version of the University of Oslo web questionnaire service “Nettskjema” demanding a governmental ID portal for login. A total of 91 patients completed their 6-month questionnaire, and of these, 50 agreed to be contacted for a possible interview. A researcher consecutively approached 21 patients, and 18 patients provided their digital consent to be interviewed.

### Data Collection

To provide the information most valuable for the study aim, an interview guide was developed based on reviewing previous research in the field, inspired by topics on innovation assessment [33], and by adding items regarding specific factors associated with the current intervention [28]. A draft for the interview guide was developed by the first author and reviewed prior to a satisfactory version for a pilot interview. No changes were made to the interview guide after the pilot interview, and the pilot interview was included in the final analysis. The interview guide (Multimedia Appendix 3) contained an introduction with questions on their use of the digital service in general, before a midpart with more detailed questions on their experience with intervention, and finally some summarizing questions allowing final reflections. The interviewer (HH) has a background as a nurse with a PhD in health service research, without any personal or professional relationship with the participants. She has conducted qualitative research and analysis earlier and led the intervention study in which this substudy arises from.

All interviews were conducted over the telephone. All participants used their private smartphones for the interviews, while the interviewer (HH) used a work-related phone, with only voice. The “Nettskjema” Dictaphone app was used to record all interviews, with 1 main recorder and a backup recorder. All recordings were satisfactory besides one, where the line was interrupted midway through, and a new recording was started. No data were lost in this recording.

All interviews were conducted between 9 AM and 5 PM, with participants offered multiple time slots to ensure convenience. The interviewer called the participants at the scheduled time, and of those consenting, only 1 never responded despite repeated attempts from the researcher. Thus, 17 interviews were conducted with consenting participants, of whom 5 had cancer, 5 had chronic pain, 4 had interstitial lung disease, and 3 had epilepsy (Table 1). All participants were interviewed only once after their 6-month follow-up, and saturation was achieved through the 17 interviews; thus, no participants were added to the interview sample. The median time from the 6-month follow-up questionnaire end to interview was 40 days (minimum-maximum 9-95 days). The interviews lasted from 15 minutes to the maximum of 46 minutes, with a median interview time of 26 minutes. Median age of the interviewed participants was 62 years (minimum-maximum 36-83 years), of whom 8 were females and 9 were males. The participants had some variation in how they had used the digital intervention, and only 1 participant was categorized as a low user (Table 2).

**Table 1:** Participants, age groups, gender, department, and interview details.

Age groups (years)	N (%)	Sex, female/male	Departments	Minutes of interview, median (minimum-maximum)
31-50	3 (17)	2/1	Neurology and pain management	27 (26-27)
51-60	4 (24)	2/2	Cancer, respiratory diseases, and neurology	24 (16-39)
61-70	6 (35)	2/4	Cancer, respiratory diseases, neurology, and pain management	22 (15-46)
71-90	4 (24)	2/2	Cancer and respiratory diseases	29 (17-33)

**Table 2:** Use of the digital intervention (N=17).

Intervention					Values
**Digital interaction^a^**					
	**Total number of digital interactions**				
			Mean (SD)	58.8 (55.6)	
			Median (minimum-maximum)	65 (3-157)	
	**Dichotomized digital interaction^b^**				
			Low use, n (%)	1 (6)	
			High use, n (%)	16 (94)	
**Asynchronous chat messages**					
	**Messages sent from patient**				
			Mean (SD)	8.4 (9.2)	
			Median (minimum-maximum)	4 (0-31)	
	**Messages sent from health care worker**				
		Mean (SD)		8.2 (7.5)	
		Median (minimum-maximum)		3 (0-20)	
	**Total number of messages**				
		Mean (SD)		16.6 (16.2)	
		Median (minimum-maximum)		12 (0-51)	
**PRO** ^c^ **measures**					
	**PRO measures sent to patient**				
		Mean (SD)		23.7 (25.4)	
		Median (minimum-maximum)		12 (0-82)	
	**PRO measure responses from patient**				
		Mean (SD)		21.4 (24.7)	
		Median (minimum-maximum)		11 (0-82)	

^a^Total counts were computed for chat messages, video visits, completed PRO measures, and monitoring events.

^b^Low users responded to less than 30% of the expected PRO measures.

^c^PRO: patient-reported outcome.

### Data Analysis

All interviews were transcribed verbatim using the F4-transcript software (Audiotranskription.de) in TSD by the first author (HH). Then, the transcripts was analyzed using thematic analysis [34], with the following steps: (1) familiarize with data, (2) generate initial codes, (3) search for themes, (4) review themes, (5) define and name the themes, and (6) produce the report. An inductive approach was used, with the research question at the outset, allowing an analysis on the premises of the data at hand [34]. Both authors read and reread the material and HH generated initial and preliminary codes of the material—interview by interview. These preliminary codes were later reviewed by EF, before they were discussed, reviewed, and refined. Through discussion, the 2 authors (HH and EF) suggested themes to describe and clarify the codes, with particular attention given to exploring how patients’ interactions with the digital health intervention may reflect aspects of their ability to access, understand, and use health information and the digital health tool according to health literacy and digital health literacy. A final set of themes, codes, and corresponding quotes was then summarized. All interviews were conducted, transcribed, and analyzed in Norwegian. The quotes have been translated to English and reviewed to assess the intended meaning. Minor alterations were made to ensure anonymity among the participants.

### Ethical Considerations

The regional ethical committee in Norway prereviewed the protocol and judged the project as outside its mandate according to the Norwegian Health Research Act (regional ethical committee south-east reference number 252051). The project was approved by the data protection officer at Oslo University Hospital (reference 21/06826) and Northern Norway University Hospital (reference 2021/4942). All participants signed a digital, written informed consent form before participating in the project. This study was conducted in accordance with the Declaration of Helsinki and all data were deidentified to ensure privacy and confidentiality. No compensation was provided to participants.

## Results

### Summary of the Themes and Codes

The thematic analysis led to 1 main theme *“*Digital outpatient care as a flexible service supporting patients’ self-management,” informed by 3 subthemes (Table 3).

**Table 3:** Themes and corresponding codes with quotes from the thematic analysis. Theme: digital outpatient care as a flexible service supporting patients’ self-management.

Subthemes and codes			Quotes
**The ongoing nature of managing a chronic condition and how the digital service meets the patients’ desire for autonomy in their care**			
	Living with a chronic condition necessitates ongoing self-management	“When you live with a diagnosis like this, it's not something you can easily forget” [P17]. “I have had some quite big seizures, and little episodes that I don’t really know whether are related to these seizures and that worries me” [P19].	
	Flexible services are required to support self-management	“It allows me to better track myself and makes me calmer in relation to the disease progression, and I believe it enables me to respond at an earlier stage than I otherwise might have if something were to happen that affects my situation. So, the motivation lies in the ability to cope with a difficult disease, I would say. There's also additional motivation in that I enjoy numbers, apps, and correlations” [P17]. “My motivation for using the app was the possibility to ask questions, and perhaps add notes to my symptoms, to be able to see some patterns” [P29].	
	Making use of digital tools for self-management supports autonomy in the patients’ role	“With this app, being able to see that the results sometimes go down a bit, and if you as a patient don't feel particularly worse, you might wait until the next measurement to make contact because you can relate this to your current situation and how you feel then, which is definitely possible with my disease” [P17]. “Sometimes, I feel I am in bad shape, but the app says I am not, and that makes me happy...because there’s so many things affecting my breath, like my state of mind and all” [P11].	
**Digital tools flexibly address the patients’ unique needs but reliability depend on patient interaction**			
	Digital tools provide new solutions to real needs in a flexible manner	“I believe that, for my part, the frequency of contact with the healthcare system has decreased since I started using the app” [P17]. “I find the app to be a part of the entire [outpatient care] system” [P36].	
	Enabling interaction through communication of unique needs beyond standardized formats	“The app asked more thorough questions than I remember being asked in person” [P12]. “You do have specific alternatives to respond to, but perhaps some lines where you could express something? It’s useful for the users, but whether it's also useful for you as the recipient, one might question, but maybe just 3-4 lines” [P36].	
	Making sure digital tools are suitable for the user	“It is indeed an advantage to have digital contact for those of us who are digital and can use such things. But it is important that the digital solutions do not replace physical attendance and postal mail, but come as an addition, that one can choose” [P6].	
**Digital services enhance the patients’ sense of safety through easy access to a relation with competent health care workers**			
	Easy and seamless contact with the outpatient clinic	“But the feeling of safety, the certainty that I could get hold of someone if there was something, that gives a lot of peace of mind for my part” [P32]. “As I am working full time, it’s a hassle to leave work to go to the clinic, and it’s adding more stress because I can’t do my job properly” [P29].	
	Digital services facilitate patient access to health care, while supporting the health care workers’ workflow	“It is a reassurance for me if I would need it, and two years ago, I had a question that I didn't know whether was important and it took a month to get an answer. If we could have done it via the app, it would have been faster for both of us instead of having to call repeatedly” [P19].	
	Building a trustful digital relation to health care workers	“Yes, I responded twice a week to the nurse and if I didn’t respond, I received a message (...) so it worked perfectly (...) but I have never spoken directly with the nurse, which by the way is exceptionally pleasant to communicate with digitally” [P22]. “I did know the doctor from before, but not the nurse – and not that it matters because now I feel like I know the nurse too, and that gives me a sense of safety” [P37].	

#### The Ongoing Nature of Managing a Chronic Condition and How the Digital Service Meets the Patients’ Desire for Autonomy in Their Care

The participants acknowledged the burden of living with a chronic condition. While some felt that they already understood the required self-management, they emphasized that digital follow-up provided a sense of support that lightened the burden, allowing them to focus more on living rather than being ill. One participant remarked, “Because I don't want to spend too much time on my condition, I want to spend my time on life instead, right? If it becomes too much, then it becomes too much concentration on the condition, and I still think that I'm not particularly sick” [P12].

The importance of self-management was highlighted, with some participants noting that this was the first time they had access to a tool that truly supported their self-management effort and the ability to monitor deterioration as highly valuable. The option to send messages, even for minor questions, was particularly appreciated, as these small clarifications provided reassurance: “Even small questions can take up a lot of space” [P34]. This access to advice enhanced their sense of control, and participants found it easier to consult health care workers through the digital platform compared with standard care.

The patients acknowledged their frequent need for health services and the challenges of contacting health care workers in outpatient care. They shared how digital outpatient care addressed this issue by improving communication and accessibility. Patients valued the efficiency and convenience offered by the digital solution, as one participant shared: “So, for me, this worked very well because I don’t need to go in just for questions. I wasn’t scared of anything, and I didn’t have to go anywhere, and I didn’t have to spend time calling, so it worked very well for me. Then you can rather go in if you don’t achieve your goals with the digital follow-up” [P29]. While the benefits of digital services were valued, the patients emphasized the need for a hybrid model with digital and physical contact. They supported innovation in health services, particularly when it contributed to flexible, individualized care and expressed positivity toward continued digital contact after the project.

Patients found the digital outpatient care tools valuable for tracking health parameters, identifying changes in their condition, thus enhancing their sense of safety. The tools were used less when their condition was stable but became essential when they suspected health changes. Through the app, patients reported gaining an understanding of their symptoms and a reflection on their condition, which they felt had an impact on their health literacy and self-management skills. In addition, the tools facilitated greater involvement of family members, providing an extra layer of support. As one patient shared: “Yes! It’s always a big moment every time I take such tests, and the people around me are always interested in how it’s going. They always ask how it went when I took these tests” [P17]. This family engagement was described as a motivating factor for using the digital platform. However, some patients noted that their use of the platform was independent of family involvement, reflecting diverse approaches to digital health engagement.

#### Digital Tools Flexibly Address the Patients’ Unique Needs, But Reliability Depends on Patient Interaction

Most patients valued the flexibility offered by digital tools, particularly the convenience of receiving care without traveling to the hospital. This was especially important for those with long travel distances or conditions that made travel burdensome. Digital tools were described as time-saving, stress-reducing, and helpful for minimizing work absences, with the ease of use and sense of security further enhancing their appeal: “If something comes up in the evening, I can enter it and then I get a response in the morning...much more frequent contact than usual” [P37]. Patients appreciated the opportunity for more flexible interactions with health care workers.

Patient-reported outcomes were generally seen as relevant and easy to complete, allowing for more thorough and individualized health reporting than traditional consultations. However, some patients felt that standardized questions and response categories did not fully capture their experiences, expressing a need for more flexibility and individual tailoring. One participant reflected: “For many of the questions I get [through the app], there are some I don’t get to elaborate enough on, and it could just be that the information that I have could be valuable to you” [P27]. Suggestions for improvement included displaying scheduled consultations and test results in the app, reducing questionnaire frequency, adding time estimates for completion, and allowing more free-text responses. Patients also expressed trust in the app’s data security but emphasized that not all data needed to be shared digitally to minimize risks.

Prerequisites for using digital tools, such as internet access, smartphones, and digital identification, were noted. Most found the app easy to navigate, with minimal training needed, although those using medical monitoring devices appreciated some initial guidance. Initial stress in using the app subsided with familiarity, as one patient noted: “At first, I was a bit stressed by it [the app], when the messages came from the hospital that I had to do these measurements, I felt that I was getting stressed. But why that is, I don’t know, it’s going well now. I was a bit surprised at myself, like, why is it stressing me, but it has passed now so that’s good because now I’m starting to get the hang of it” [P11]. However, digital tools were not universally suitable, with concerns raised about older adult users, individuals with health-related anxiety, and the timing of app prompts for working patients. Despite these concerns, reminders were often described as helpful for completing tasks.

#### Digital Services Enhance the Patients’ Sense of Safety Through Easy Access to a Relation With Competent Health Care Workers

The patients appreciated the sense of security offered by the user-friendly and flexible digital contact with health care workers in outpatient care. The app was seen as supportive rather than burdensome, with some noting that spending a few minutes weekly was a small effort for managing a long-term condition: “It’s not like the app reminds you that you have a chronic condition, it’s more perceived as a positive element in addition to the treatment you’re already receiving” [P17]. Digital reporting was practical and reduced errors, although patients cautioned against relying on technology for false security.

Avoiding phone queues and callbacks saved time and reduced stress for patients, while also improving workflow for health care workers by allowing responses on their schedule. One participant shared: “I liked that I seemed to have better access to the department than normal, it was less burdensome than calling in the limited time the reception is open, and sometimes it rings and rings and rings and rings, and sometimes dealing with less or even unqualified reception workers which I have had problems with before” [P19]. Asynchronous messaging was particularly valued for enabling prompt answers to both urgent and nonurgent questions, fostering a sense of being seen and supported. Even when unused, the messaging function reassured patients that they could reach skilled health care workers if needed.

The competence of health care workers responding to queries was trusted, and while some patients valued consistent interactions with the same person, others prioritized competence over familiarity, especially with nurses. Patients appreciated building relationships through physical consultations before transitioning to digital follow-up but found digital contact effective once trust was established, as one patient elaborated: “And yes, I’ve met them, they know me and I have a face to them, and I think that’s very good. That it’s the same people who follow up. But it’s mostly on the doctor's side that I need to have the same person, whom I trust, and I have met in consultations, who knows me. But with the nurses, I think it’s very good to have two people” [P14]. Administrative workers triaging questions were considered acceptable, provided medical issues were handled by qualified professionals. However, some patients still preferred direct calls to familiar health care workers, especially if they already had access to adequate resources.

## Discussion

### Principal Findings

The patients experienced digital outpatient care as a flexible service supporting self-management, which fulfilled the patients’ ongoing but unmet need of autonomy in their self-management. The findings suggest that aspects of health literacy, such as the ability to understand, engage with, and act on health information, may have influenced how patients interacted with the digital platform and benefited from the service. To achieve a truly flexible and reliable service, active interaction between patients and health care workers with the digital platform is essential to strengthen the patients’ sense of safety. The patients’ reflections regarding the digital outpatient service in this study highlight key insights worth further discussion.

From the patients’ perspective, a digital outpatient solution offers greater flexibility in health care services than traditional calendar-based approaches, which rely solely on phone calls or scheduled consultations to access the outpatient clinic. Previous research has described how traditional systems are rigid and allow little patient-centered and flexible approaches [35]. If changes in the patients’ clinical status occur, there is little room for a rapid response or to move forward a scheduled appointment to curb the deterioration. However, the digital opportunities include flexibility through asynchronous messaging, monitoring, and patient responses—offering a more patient-centered approach, supporting the patient’s self-management [28]. Furthermore, health literacy is often intertwined with self-management, and both concepts are positively and iteratively influencing each other [16,31]. This highlights how digital outpatient solutions not only enhance the patients’ experience of more flexibility and responsiveness but also foster patient-centered care by supporting self-management and strengthening health literacy in a mutually reinforcing manner.

While our study and similar research highlight the benefits of digital approaches [5,10-12,18], some patients may feel burdened by the added responsibility, potentially threatening their sense of security. One patient described initial anxiety when using the digital platform, fearing mistakes in reporting—a concern echoed in previous studies emphasizing the critical role of usability and adequate digital and health literacy [36,37]. However, with practice, participants gained confidence in self-management and their sense of insecurity decreased. Interaction with health care workers further supported this transition, providing timely guidance and encouragement through digital messaging. This highlights the importance of tailoring support to help reluctant patients build the necessary skills and competencies for digital outpatient care [5,18]. In our study, patients demonstrated health literacy through their engagement with the intervention, responding accurately to patient-reported outcome measures, conducting remote monitoring as instructed, and framing thoughtful questions via the messaging functions, showcasing the integral link between health literacy and effective self-management.

The flexibility and interactive opportunities provided by the digital platform highlight its potential to reduce the traditional asymmetry between patients and health care providers. Unlike calendar-based systems in traditional care, which often overlook individual patient needs, digital outpatient care allows patients to engage with health care workers when changes in their clinical condition occur. Over time, patients became more adept at interpreting the relationship between their symptoms and objective measurements provided by the system, enabling them to make informed decisions and take appropriate actions with guidance from health care workers [28]. In the digital solution, where interactions occurred based on asynchronous messages, the focus of the interaction was more centered on the patients’ input and experiences rather than being primarily driven by the health care workers [6]. The role of the health care worker is also evolving, much like the changes digital solutions bring to the patient’s role. More flexible ways of interacting with patients are shifting the responsibilities of health care workers, often in ways they are not fully prepared for. However, with the necessary competencies in using digital solutions and a clear understanding of their integration into clinical care, health care workers can positively influence patients’ engagement and effective use of digital health solutions to maintain or improve health [38,39]. While processes that strengthen the patient role are well established in patient-centered care [6], their implementations through digital solutions remain underexplored.

Based on our findings, we argue that digital outpatient care can enhance care quality by providing easier and more timely access to health services, saving time and potentially preventing symptom deterioration through early identification of changes. Patients’ trust in the system and providers, their increased self-management, and reduced feelings of burden—both as individuals living with a chronic condition and as users of health care services—further support this claim. However, the impact of digital outpatient care on health outcomes related to patients’ conditions remains unclear. While previous research has shown positive but small and low-graded evidence on such effects [5,7,18], further studies are needed to evaluate both health outcomes and the experiences of patients and health care providers using integrated digital health solutions.

### Limitations and Strengths

This study is among the few qualitative investigations into integrated digital health solutions in outpatient care, but it has limitations. Participants were recruited from a study evaluating the same digital platform, with tailored interventions based on clinical needs, leading to heterogeneous experiences. All interviews were conducted using telephone with only voice, and thus, potentially relevant data concerning body language and nonverbal communication were not interpreted. To address this, a semistructured interview guide was used, and while data analysis was comprehensive, recurring similar statements were interpreted as having broader relevance. A key finding was that all patients expressed trust in the digital solution and care provided. However, this may introduce bias, as participants were likely those already comfortable with digital solutions, potentially excluding perspectives from less digitally inclined individuals.

### Conclusions

In this study, we aimed to explore the experiences of patients with long-term conditions in using a digital outpatient care service for 6 months. Digital outpatient care was experienced as flexible and supportive for patients with long-term conditions. The increased possibility of interacting with health care workers was welcomed by the patients, and the combination of flexibility, self-monitoring, and addressing concerns regarding their self-management seemed to increase the patients’ experience of autonomy. As health literacy likely plays a role in patients’ ability to effectively engage with digital tools and self-manage their conditions, future research should explore how varying levels of health literacy influence these outcomes. In addition, research should address whether such digital outpatient clinics are positive for a wider range of patients, associated health outcomes, and any positive effects on a health system level.

## Appendix

Multimedia Appendix 1 Dignio Prevent interface.

Multimedia Appendix 2 MyDignio interface.

Multimedia Appendix 3 Interview guide.

Multimedia Appendix 4 COREQ (Consolidated criteria for Reporting Qualitative research) checklist.

## Abbreviations

COREQ: Consolidated criteria for Reporting Qualitative research
TSD: Tjeneste for Sensitive Data

## Data Availability

The datasets used and analyzed during this study are available from the corresponding author on reasonable request.
