# Supplementary material for: “I Want to Spend My Time Living”—Experiences With a Digital Outpatient Service With a Mobile App for Tailored Care Among Adults With Long-Term Health Service Needs: Qualitative Study Using Thematic Analysis
Source: J Med Internet Res. 2026 Jan 15;28:e79155. doi: 10.2196/79155 (PMC12856408; doi:10.2196/79155)
Supplement: Multimedia Appendix 1 [file jmir_v28i1e79155_app1.docx]

# Multimedia Appendix 1. Dignio Prevent dashboard.

# Holmen and Fosse—a thematic analysis of digital outpatient services for adults.

Previously published: Holmen H, Holm AM, Kilvær TK, Ljoså TM, Granan LP, Ekholdt C, et al. Digital outpatient services for adults: development of an intervention and protocol for a multicenter non-randomized controlled trial. *JMIR Res Protoc*. Jul 10, 2023;12:e46649.


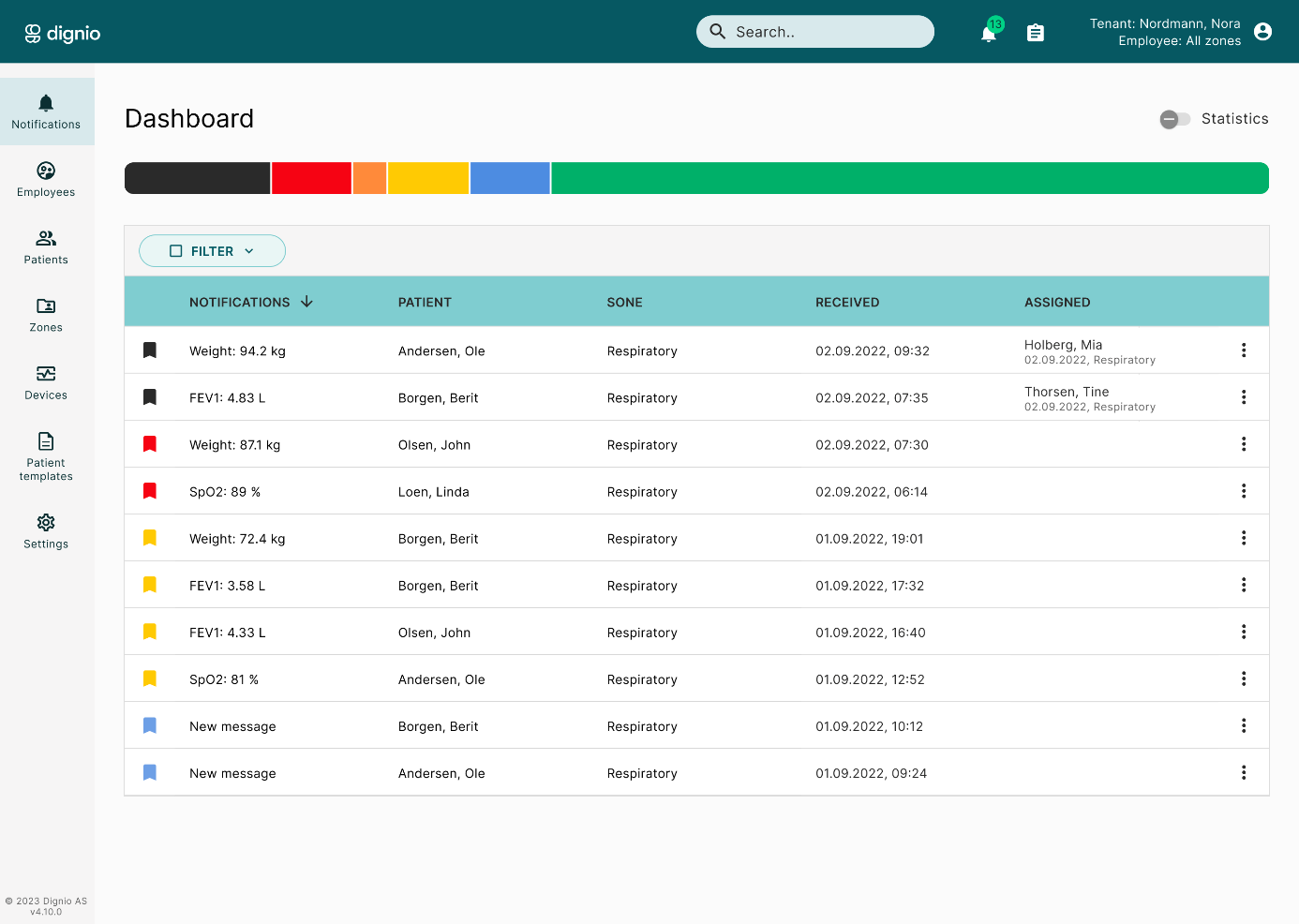


Appendix Figure 3. Dashboard in Dignio Prevent, as the health care workers see it. All names, dates, and values are repoduced and do not contain real patient or health care worker data.
