# Supplementary material for: “I Want to Spend My Time Living”—Experiences With a Digital Outpatient Service With a Mobile App for Tailored Care Among Adults With Long-Term Health Service Needs: Qualitative Study Using Thematic Analysis
Source: J Med Internet Res. 2026 Jan 15;28:e79155. doi: 10.2196/79155 (PMC12856408; doi:10.2196/79155)
Supplement: Multimedia Appendix 2 [file jmir_v28i1e79155_app2.docx]

# Multimedia Appendix 2. MyDignio.

# Holmen and Fosse—a thematic analysis of digital outpatient services for adults.

Previously published: Holmen H, Holm AM, Kilvær TK, Ljoså TM, Granan LP, Ekholdt C, et al. Digital outpatient services for adults: development of an intervention and protocol for a multicenter non-randomized controlled trial. *JMIR Res Protoc*. Jul 10, 2023;12:e46649.


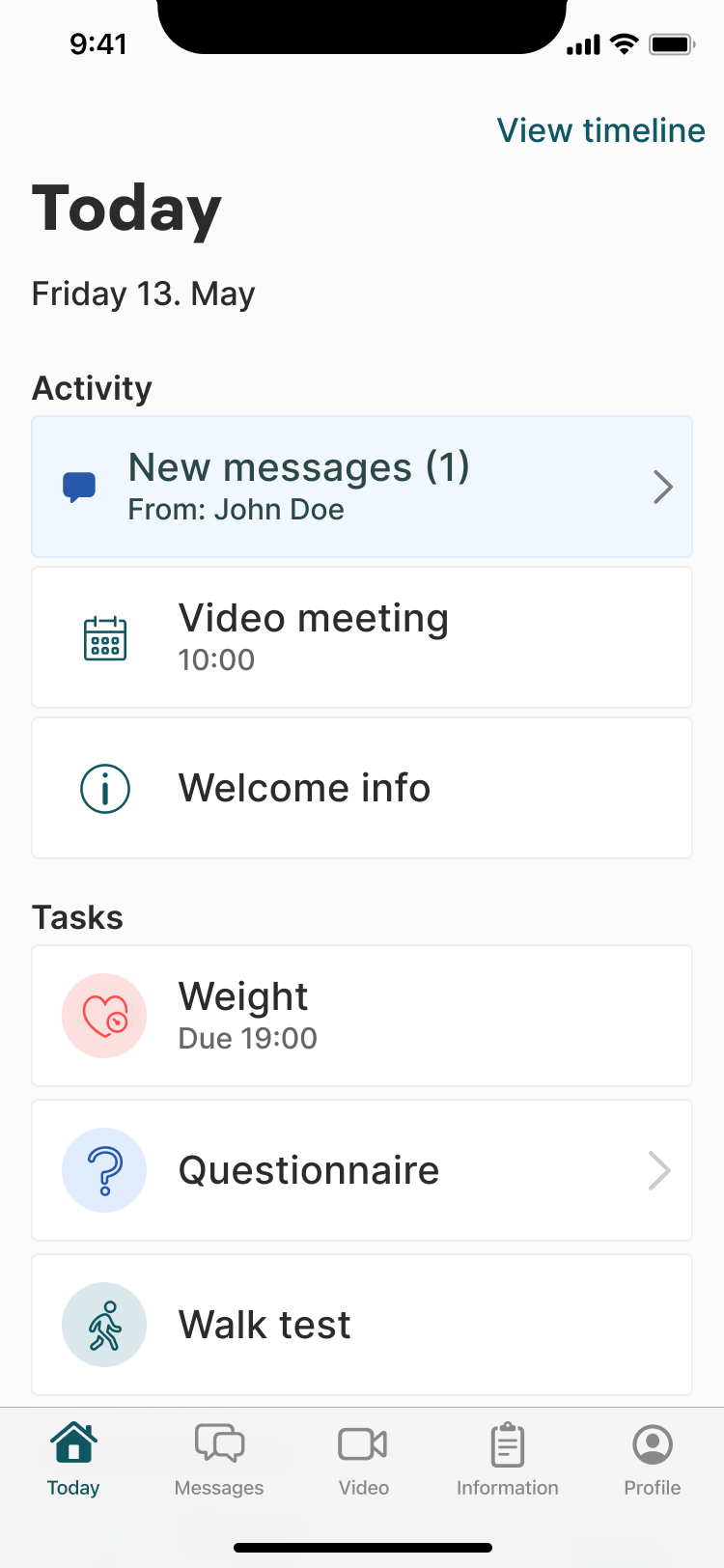


Appendix Figure 4. Screen of MyDignio, as the patients’ see it. All names, dates, and values are repoduced and do not contain real patient data.
