# Supplementary material for: “I Want to Spend My Time Living”—Experiences With a Digital Outpatient Service With a Mobile App for Tailored Care Among Adults With Long-Term Health Service Needs: Qualitative Study Using Thematic Analysis
Source: J Med Internet Res. 2026 Jan 15;28:e79155. doi: 10.2196/79155 (PMC12856408; doi:10.2196/79155)
Supplement: Multimedia Appendix 3 [file jmir_v28i1e79155_app3.docx]

# Multimedia Appendix 3. Interview guides.

# Interview guide, Holmen and Fosse—a thematic analysis of digital outpatient services for adults.

Previously published: Holmen H, Holm AM, Kilvær TK, Ljoså TM, Granan LP, Ekholdt C, et al. Digital outpatient services for adults: development of an intervention and protocol for a multicenter non-randomized controlled trial. *JMIR Res Protoc*. Jul 10, 2023;12:e46649.

## Interview guide to patients

Introduction

Briefly about the purpose and goals of the study and the complementary interviews. The aim of this interview is to delve deeper into patients' experiences of using a digital solution for follow-up and communication with healthcare staff in outpatient clinics. You are asked to take part in an interview because you have taken part in the project where MyDignio has been tested and you have agreed that we can interview you.

The interviewer present herself and allow the interviewee to do the same, before going into the more specific questions in the semi-structured guide.

Details related to the intervention:

1. Which parts of the MyDignio app do you use/have you used?
2. Do you feel that follow-up through MyDignio differs from normal outpatient consultations? How/how not?
3. Do you have relatives who contribute to your treatment and follow-up?
   1. If so, to what extent do they contribute?

Focus areas in the coming questions: (feeling of security, well-being, sense of mastery, control over one's own health, motivation and activity level.)

1. Compared to previous treatment options, what degree of security did you think MyDignio offers you?
   1. How would you change follow-up through MyDignio if you could?
2. Do you enjoy follow-up through MyDignio?
   1. What do you like/dislike?
3. Did you receive any training initially? If so, how was the training useful?
   1. Was there any specific part of MyDignio that was difficult to understand?
4. Do you feel that MyDignio has given you greater control and understanding of your own health?
   1. What would you like differently?
5. Are you motivated to use MyDignio?
   1. What contributes to this / prevents this?
6. How has your daily activity level changed as a result of follow-up through MyDignio?
   1. What new activities do you have the opportunity to undertake as a result of this?
   2. Do you feel that this contributes to well-being and improving health?

Concluding:

1. What is your overall experience with MyDignio?
2. Is there anything you miss?
3. Is there anything you would like to see differently?
4. Something you want to say that you haven't been able to say?

## Interview guide to healthcare workers

Introduction

Briefly about the purpose and goals of the study and the interviews.

The interviewer introduce herself, and allow the interviewee to do the same, before going into the more specific questions in the semi-structured guide.

Details related to the intervention:

1. Which of the various components of DignioPrevent have your patients used?
2. How do you feel that follow-up through DignioPrevent differs from regular outpatient consultations? (safety, infection, number of consultations and practical implementation)
3. Do you have examples where relatives have also contributed to treatment and follow-up?
   1. How?
4. How would you change follow-up through DignioPrevent if you could?
5. Do you enjoy follow-up through DignioPrevent?
   1. What do you like/dislike?
6. How did you experience the initial training?
   1. Was there any specific part of DignioPrevent that was difficult to understand?
7. Do you feel that DignioPrevent and MyDignio give patients greater control and understanding of their own health?
   1. Anything you wish were different?
8. What do you think the patients would want differently?
9. Are you motivated to use DignioPrevent?
   1. What contributes to this / prevents this?
10. How has DignioPrevent changed the workflow in your everyday life?
    1. Does this also apply to other working groups?
    2. do you have examples where healthcare personnel have been able to follow up patients from home?

Concluding:

What is your overall experience with DignioPrevent?

Is there anything you miss?

Is there anything you would like to see differently?
